# Cytoskeletal Dynamics and Molecular Motor Dysfunction in Psychiatric Disorders: Insights from Schizophrenia and Autism Spectrum Disorder

**DOI:** 10.3390/biology15070550

**Published:** 2026-03-30

**Authors:** Kenyu Nakamura, Asumi Kubo, Sae Sanaka, Sara Kamiya, Kentaro Itagaki, Tetsuya Sasaki

**Affiliations:** 1 Laboratory of Anatomy and Neuroscience, Department of Biomedical Sciences, Institute of Medicine, University of Tsukuba, 1-1-1 Tennodai, Tsukuba 305-8577, Ibaraki, Japan; 2 College of Medicine, School of Medicine and Health Sciences, University of Tsukuba, 1-1-1 Tennodai, Tsukuba 305-8577, Ibaraki, Japan; 3 Master’s Program in Frontier Medical Sciences, Degree Program of Comprehensive Human Sciences, Graduate School of Comprehensive Human Sciences, University of Tsukuba, 1-1-1 Tennodai, Tsukuba 305-8577, Ibaraki, Japan; 4 College of Medical Sciences, School of Medicine and Health Sciences, University of Tsukuba, 1-1-1 Tennodai, Tsukuba 305-8577, Ibaraki, Japan; 5 PhD Program of Neurosciences, Degree Program of Comprehensive Human Sciences, Graduate School of Comprehensive Human Sciences, University of Tsukuba, 1-1-1 Tennodai, Tsukuba 305-8577, Ibaraki, Japan

**Keywords:** cytoskeleton, molecular motors, schizophrenia, autism spectrum disorder, microglia

## Abstract

Elucidating the pathophysiological mechanisms of mental disorders remains a critical challenge in psychiatric research. Recent studies have highlighted the potential involvement of cytoskeletal and molecular motor abnormalities in the development of mental disorders such as schizophrenia and autism spectrum disorder (ASD). Although schizophrenia and ASD differ clinically, both disorders are increasingly regarded as neurodevelopmental conditions and share vulnerabilities in synapse formation and neural circuit maturation. This review synthesizes the latest findings on the relationship between cytoskeletal and molecular motor abnormalities and mental disorders. The cytoskeleton, composed of microtubules, actin filaments, and intermediate filaments, along with molecular motors such as kinesins, dyneins, and myosins, plays crucial roles in neurodevelopment, synapse formation, and neurotransmission. In schizophrenia, decreased expression of the microtubule-associated protein MAP2 and abnormalities in the DISC1 gene have been reported, potentially leading to dendritic morphological abnormalities and neurodevelopmental disorders. Additionally, abnormalities in molecular motors such as KIF17 and KIF1A have been implicated in schizophrenia pathophysiology. Myosin Id has been identified as a risk gene for ASD. Furthermore, abnormalities in actin-related proteins such as SHANK3 and CYFIP1 have been shown to cause synaptic dysfunction. These findings suggest that mental disorders arise from complex pathologies involving multiple cytoskeletal and molecular motor-related protein abnormalities. Future research should focus on elucidating the functions of individual proteins and adopting a comprehensive approach that includes glial cells. Advances in this field may deepen our understanding of the pathophysiological mechanisms of mental disorders and potentially lead to the development of novel therapeutic strategies.

## Simple Summary

This review provides a concise overview of how abnormalities in cytoskeletal proteins and molecular motors contribute to psychiatric disorders, focusing on schizophrenia and autism spectrum disorder (ASD). The cytoskeleton and molecular motors are essential for neuronal structure and communication, and disruptions in these systems—particularly in kinesin, myosin, and actin-related proteins—are increasingly linked to disease mechanisms in both conditions. We also highlight the emerging role of microglial cytoskeletal remodeling as a neuroimmune contributor to psychiatric pathophysiology. These findings point toward novel therapeutic targets in cytoskeletal and motor pathways.

## 1. Introduction

Neurons possess a highly organized cytoskeletal system that enables them to maintain their unique morphology and specialized functions. This cytoskeletal network, composed primarily of microtubules, actin filaments, and intermediate filaments, plays essential roles in numerous cellular processes, including neuronal development, axonal and dendritic outgrowth, and synapse formation [1]. In addition, molecular motors—particularly members of the kinesin superfamily (KIFs)—travel along these cytoskeletal tracks to transport various intracellular cargos, thereby contributing critically to the maintenance of neuronal function [2,3].

Recent research has revealed that abnormalities in cytoskeletal structures and molecular motor proteins are deeply implicated in the pathophysiology of several psychiatric disorders, most notably schizophrenia and autism spectrum disorder (ASD) [4,5]. In this review, we summarize the fundamental functions of the cytoskeleton and molecular motors, and we discuss how disruptions in these systems contribute to psychiatric disease mechanisms, with a particular focus on schizophrenia and ASD.

Polygenic risk is enriched in genes involved in neurodevelopment and synaptic function, and increasing evidence implicates immune dysregulation and neuroinflammation (e.g., maternal immune activation and microglial activation) as modulators of circuit maturation [6,7]. These shared features provide a framework for examining how cytoskeletal regulation and intracellular transport shape synapse development, receptor trafficking, and network-level function. Schizophrenia is associated with cortical thinning, ventricular enlargement, and widespread functional dysconnectivity, particularly affecting prefrontal–temporal networks involved in cognitive control and working memory. In contrast, ASD has been associated with early brain overgrowth during childhood, alterations in cortical surface area development, and atypical functional connectivity within social brain networks and the default mode network [8].

Schizophrenia and ASD differ substantially in developmental trajectory and symptom profile: ASD presents in early childhood with persistent social-communication impairments and restricted/repetitive behaviors, whereas schizophrenia typically emerges in late adolescence with psychosis and cognitive disturbances [4,5]. Both disorders are highly heterogeneous, with diverse genetic architectures and environmental influences; cytoskeletal and motor dysfunction likely represents one of several converging biological pathways contributing to disease pathophysiology.

The neuronal cytoskeleton provides both structural support and dynamic scaffolding for morphogenesis and plasticity. Microtubules support axonal/dendritic growth and serve as tracks for long-range transport [9,10,11] and intermediate filaments contribute to cellular stability and axonal caliber [12,13]. Perturbations in these systems can therefore impact neuronal polarity, arborization, and synaptic architecture—cellular phenotypes repeatedly implicated in schizophrenia and ASD.

Molecular motors convert ATP hydrolysis into directed movement along cytoskeletal filaments. Kinesins primarily drive anterograde transport on microtubules, dynein mediates retrograde transport, and myosins regulate actin-based trafficking and spine dynamics [14,15,16]. By controlling the spatiotemporal delivery of receptors, vesicles, organelles, and mRNAs, these motors directly influence synaptogenesis, synaptic plasticity, and circuit maturation, providing mechanistic links between genetic/molecular perturbations and disease-relevant cellular phenotypes.

Increasing genetic and molecular evidence indicates that specific cytoskeletal and molecular motor proteins are directly linked to psychiatric disorders. For example, alterations in kinesin family members (such as KIF1A, KIF5C, and KIF17), dynein components (DYNC1H1), and actin-associated motors (MYO16 and MYO1D) have been reported in individuals with schizophrenia or ASD. These proteins regulate intracellular trafficking of synaptic vesicles, neurotransmitter receptors, and signaling complexes, suggesting that disruption of intracellular transport may contribute to synaptic dysfunction and abnormal circuit development [4,5,17].

Kinesin superfamily proteins (KIFs) mediate anterograde transport toward microtubule plus-ends, delivering cargos from the soma to axons and dendrites [18]. More than 45 KIF genes have been identified in humans, with individual KIFs transporting distinct cargo sets including synaptic vesicles, neurotransmitter receptors, mitochondria, and mRNA granules [18,19,20,21,22].

Among kinesins, KIF5A/B/C (kinesin-1 family) mediate long-range anterograde transport of mitochondria, membranous organelles, and receptor-containing vesicles, while KIF1A transports synaptic vesicle precursors and KIF17 delivers NMDA receptor subunits to dendrites [18,23].

Dynein, in contrast, mediates retrograde transport toward the minus-end of microtubules, moving cargos from axonal terminals back toward the cell body [24]. Its major roles include the retrograde trafficking of synaptic vesicles, neurotransmitter receptors, and various organelles; the transport of neurotrophic factor signaling endosomes; and the redistribution of cellular components during axon regeneration [25,26,27,28].

Myosins function primarily along actin filaments [29]. Numerous myosin family members are expressed in neurons, where they regulate the transport of diverse cargos and modulate actin architecture to influence synaptic activity. Myosins contribute to dendritic spine morphogenesis, local mobility of synaptic vesicles, growth cone motility, and the regulation of synaptic plasticity [30,31,32,33]. Myosin II, V, and VI play essential roles in neuronal physiology.

Together, these molecular motor systems coordinate the highly dynamic intracellular transport processes required for neuronal development, synaptic function, and circuit maintenance. Dysfunction in any of these motor proteins can profoundly disrupt neuronal connectivity and is increasingly recognized as a key contributor to neurodevelopmental and psychiatric disorders [1,23].

Psychiatric disorders such as schizophrenia and ASD impose a substantial societal burden, yet their molecular–cellular underpinnings remain incompletely understood. While synaptic and neurodevelopmental frameworks have been extensively discussed, the cytoskeleton and intracellular transport machinery—which directly govern neuronal morphogenesis, cargo trafficking, and synaptic organization—are often treated as background cell biology rather than as central disease mechanisms. The present review is a narrative review based on studies identified through PubMed and Google Scholar, using search terms including cytoskeleton, molecular motors, kinesin, dynein, myosin, schizophrenia, and autism spectrum disorder, primarily covering the period from 2000 to the present. Studies were prioritized based on the provision of (i) genetic evidence (e.g., GWAS, rare variants, copy-number variants), (ii) human brain evidence (e.g., postmortem expression or proteomics), and/or (iii) mechanistic support from cellular or animal models linking a specific cytoskeletal or motor protein alteration to a neuronal or glial phenotype relevant to disease. No formal systematic-review guidelines were applied; instead, the review focused on mechanistically informative and recent evidence, summarized in Table 1. The novelty of this review lies in its integration of foundational cytoskeletal biology with evidence from recent large-scale genomic studies, including a genome-wide association study of schizophrenia identifying 287 loci with convergence on synaptic and cytoskeletal gene networks [34], and a large-scale exome-sequencing study in ASD implicating more than 100 risk genes enriched in neurodevelopmental pathways [35]. These converging lines of genomic evidence substantially reinforce cytoskeletal and motor dysfunction as a shared pathological axis in both disorders. Furthermore, this review reflects the research interests of our laboratory, which has been investigating the roles of molecular motors and microtubule-associated proteins in neuronal function and psychiatric disease. Our group has demonstrated that Myosin Id accumulates in dendritic spines through its TH1 domain [36] and functions as a risk gene for ASD, and has shown that KIF17-mediated transport of NMDA receptor subunits is regulated in an activity-dependent manner [37]. In addition, our recent work has revealed that the expression of psychiatric disorder-related kinesin superfamily proteins is upregulated in alternatively activated microglia [38], suggesting a neuroimmune dimension to motor protein dysregulation. This review therefore integrates our laboratory’s findings with the broader field to provide a perspective on cytoskeletal and molecular motor dysfunction as a convergent and therapeutically relevant mechanism in schizophrenia and ASD.

## 2. Cytoskeletal and Molecular Motor Abnormalities in Schizophrenia

Table 1 summarizes representative cytoskeletal and molecular motor genes implicated in schizophrenia and ASD, together with reported variants, molecular functions, and proposed cellular consequences.

Schizophrenia is a psychiatric disorder characterized by hallucinations, delusions, cognitive impairments, and disturbances in emotion and behavior. Accumulating evidence indicates that abnormalities in cytoskeletal components and molecular motor proteins are present in the brains of individuals with schizophrenia and are closely linked to disease pathophysiology.

Among microtubule-related abnormalities, reduced expression of the microtubule-associated protein MAP2 has been reported in the prefrontal cortex and hippocampus of patients with schizophrenia [39,40]. Because MAP2 plays a critical role in the formation and maintenance of dendrites, decreased expression is thought to contribute to dendritic morphological abnormalities and impaired dendritic function. In addition, DISC1 (Disrupted in Schizophrenia 1), a susceptibility gene for schizophrenia, regulates microtubule stabilization and intracellular transport. Dysfunction of DISC1 is believed to impair neurodevelopment and synaptic plasticity [41,42]. Among the reported variants, the missense single nucleotide polymorphism rs821616 (p.Ser704Cys) in DISC1 is among the best-studied; this variant disrupts the interaction between DISC1 and its binding partners NDEL1 and LIS1, which are essential for microtubule stabilization and nuclear migration during cortical development. Additional SNPs, including rs1000731, have been associated with reduced hippocampal volume and impaired working memory in schizophrenia cohorts.

Abnormalities in actin-related proteins have also been identified in schizophrenia. Postmortem studies have reported altered expression of components of the WAVE complex—such as CYFIP1, NCKAP1, and WASF1 [43]—which regulates actin polymerization and contributes to dendritic spine formation. Disruption of this complex is expected to lead to synaptic dysfunction [44]. At the genetic level, 15q11.2 microdeletions—which encompass CYFIP1—represent one of the most frequent copy-number variants (CNVs) associated with schizophrenia (OR ≈ 2.8), causing ~50% reduction in CYFIP1 protein levels. This haploinsufficiency impairs assembly of the WAVE regulatory complex and Rac1-mediated actin polymerization, resulting in reduced density and abnormal morphology of dendritic spines [43,44]. Increased expression of calponin-3, an actin-binding protein, has also been observed in the prefrontal cortex of patients [47]. Elevated levels of calponin-3 may contribute to aberrant stabilization of the actin cytoskeleton [48].

Regarding intermediate filaments, increased expression of GFAP (glial fibrillary acidic protein) has been detected in the prefrontal cortex and hippocampus of individuals with schizophrenia [49]. As a major intermediate filament protein in astrocytes, elevated GFAP expression likely reflects neuroinflammatory or neuroprotective responses [50].

Genetic studies have suggested that variants affecting the KIF1A pathway may influence synaptic vesicle transport mechanisms relevant to psychiatric disorders. Because KIF1A mediates the anterograde transport of synaptic vesicle precursors, its dysfunction may reduce synaptic transmission efficiency and alter neuronal connectivity. Regarding KIF17, the missense single nucleotide polymorphism rs2722519, together with reduced KIF17 protein expression, has been reported in schizophrenia patients [51,52]. This variant impairs anterograde transport of NR2B-containing NMDA receptors to synapses, disrupting synaptic plasticity. Genetic association studies have also identified single nucleotide polymorphisms within KIF1A, including variants in the motor and stalk domains [53,54], predicted to reduce microtubule-based processivity and diminish anterograde delivery of synaptic vesicle precursors. In addition, a mutation in KIF3B has been shown to reduce trafficking of the NMDA receptor subunit NR2A and to cause schizophrenia-like behavioral phenotypes in mice [57]. Genetic studies have suggested potential links between KIF1A variants and synaptic vesicle transport deficits relevant to schizophrenia [54], with disruption of KIF1A processivity proposed as a contributing mechanism.

Dynein-related abnormalities include rare variants in the DYNC1H1 (Dynein Cytoplasmic 1 Heavy Chain 1) gene identified in individuals with schizophrenia [58]. Because DYNC1H1 is a major component of the dynein complex, its dysfunction is expected to impair retrograde axonal transport [59]. Variants in MYO16 (Myosin XVI), a myosin family protein involved in neuronal migration and dendrite formation, have also been reported in schizophrenia [60]. Disruption of MYO16 function may impair neural circuit formation. Specifically, de novo missense variants such as p.His306Arg in DYNC1H1, documented in patients with neurodevelopmental disorders [58,59], are predicted to destabilize the dynein motor domain, impairing retrograde trafficking of BDNF-containing signaling endosomes and compromising neurotrophin-dependent neuronal survival signaling. Variants in MYO16 (Myosin XVI) associated with schizophrenia include rare missense and copy-number variants identified in Han Chinese GWAS cohorts [60]. MYO16 is expressed in postmitotic neurons and regulates cytoskeletal dynamics during neuronal migration and dendrite morphogenesis; disruption of MYO16 function is therefore expected to contribute to circuit formation abnormalities observed in schizophrenia.

Collectively, these cytoskeletal and molecular motor abnormalities contribute to a range of pathological processes observed in schizophrenia, including impaired synapse formation and plasticity, abnormal receptor trafficking, disrupted neurodevelopment, dysfunctional neural circuits, and cognitive deficits. These findings underscore the crucial role of intracellular transport and cytoskeletal regulation in the neurobiology of schizophrenia.

## 3. Cytoskeletal and Molecular Motor Abnormalities in Autism Spectrum Disorder

Autism spectrum disorder (ASD) is a neurodevelopmental condition characterized by impairments in social communication and interaction, together with restricted, repetitive patterns of behavior and interests. In recent years, multiple abnormalities in cytoskeletal components and molecular motor proteins have been reported in the brains of individuals with ASD.

Myosin Id (MYO1D) has been identified as a risk gene for ASD [17]. We previously showed that EGFP-tagged Myosin Id, when expressed in cultured neurons, accumulates in dendritic spines [36]. Furthermore, this localization critically depends on the TH1 (tail homology 1) domain: deletion of the TH1 domain disrupts dendritic targeting and markedly reduces its accumulation in dendritic spines. These findings strongly suggest that MYO1D interacts with actin filaments within dendritic spines and contributes to the regulation of excitatory synaptic transmission, and that disruption of this function may underlie aspects of ASD pathophysiology. The TH1 domain of MYO1D has also been reported to interact with the C-terminus of aspartoacylase, an N-acetylaspartate (NAA)–acylating enzyme [62]. NAA is present at high concentrations in the mammalian brain, and reduced NAA levels have been observed in children with ASD compared with typically developing controls [63]. These observations further support a functional link between MYO1D and ASD.

Myosin IXb (MYO9B) is known to regulate RhoA activity and to control dendritic morphology in cortical neurons [64]. Variants in MYO9B have been suggested to influence ASD risk [5]. Rare missense variants in MYO9B affecting its RhoGAP domain have been identified in ASD patients; these variants are predicted to impair RhoA inactivation, leading to dysregulation of actin dynamics and dendritic arborization [64]. In addition, cohort studies have reported an association between Myosin XVI and ASD [61]. Myosin XVI contributes to neuronal migration and dendritic development, and its dysfunction is thought to lead to defects in neural circuit formation.

Mutations in SHANK3 (SH3 and multiple ankyrin repeat domains 3) are recognized as major genetic risk factors for autism spectrum disorder (ASD) [65]. SHANK3 plays a key role in organizing the actin cytoskeleton in the postsynaptic density; its disruption leads to defective dendritic spine formation and synaptic dysfunction [66]. Duplications and deletions of CYFIP1 (Cytoplasmic FMR1-interacting protein 1) have also been identified as risk factors for ASD [45]. CYFIP1 is a component of the WAVE complex and regulates actin polymerization; its dysregulation is believed to cause dendritic spine abnormalities [46]. Specifically, heterozygous deletions of chromosome 22q13.3 encompassing SHANK3, as well as truncating and frameshift mutations have been identified in individuals with ASD and Phelan-McDermid syndrome [65]. These loss-of-function variants lead to near-complete absence of SHANK3 at the postsynaptic density, resulting in severely impaired actin cytoskeleton organization and dendritic spine morphogenesis.

Rare variants in TUBG1 (γ-tubulin) have been reported in individuals with ASD [67]. γ-Tubulin functions as a nucleation factor for microtubule assembly, and its disruption is expected to affect neuronal polarity and migration [68]. Mutations in ASPM (abnormal spindle-like microcephaly-associated protein) have likewise been implicated as risk factors for ASD [69]. ASPM plays an important role in the division and differentiation of neural stem cells, and its dysfunction is thought to lead to abnormal cortical development [70]. Biallelic loss-of-function mutations in ASPM—including frameshift and nonsense variants distributed throughout the IQ (isoleucine-glutamine) calmodulin-binding motif repeats—cause autosomal recessive primary microcephaly; heterozygous rare variants have been identified in ASD cohorts and are thought to impair the symmetric division of neural progenitors, reducing the final neuronal output of the developing cortex [69,70]. The de novo missense variant p.Tyr92Cys in TUBG1, reported in individuals with lissencephaly-spectrum cortical malformations and ASD-associated features [67,68,71], disrupts the γ-tubulin ring complex, impairing microtubule nucleation at the centrosome and Golgi apparatus and thereby compromising neuronal polarity and radial migration.

Rare variants in KIF1A have been identified in patients with ASD [55]. KIF1A is essential for the anterograde transport of synaptic vesicle precursors, and its dysfunction is predicted to reduce the efficiency of synaptic transmission [56]. Mutations in KIF5C have also been associated with ASD. KIF5C contributes to neuronal polarity and axonal elongation, and its disruption likely impairs neural circuit formation [71]. Among the reported KIF5C mutations, the de novo missense variant p.Glu237Lys, identified in patients with malformations of cortical development and intellectual disability [71], is located within the kinesin motor domain and disrupts ATP hydrolysis, severely impairing microtubule-based anterograde transport and thereby causing abnormal axon elongation and cortical wiring defects. Rare de novo missense variants including c.773C > T (p.Pro258Leu) and other motor domain mutations have been identified in ASD patients [55,56]. These variants impair the ATPase activity and processivity of KIF1A, leading to insufficient anterograde delivery of dense-core vesicles and synaptic vesicle precursors to distal axons and dendrites.

Taken together, these cytoskeletal and molecular motor abnormalities appear to contribute to ASD pathophysiology through several interrelated mechanisms. First, disturbances in actin-regulating proteins such as SHANK3 and CYFIP1 result in defective dendritic spine formation and synaptic dysfunction, thereby disrupting the normal development and function of neural circuits. Second, abnormalities in kinesins are expected to impair axonal transport and neuronal migration, leading to structural and functional abnormalities in neural networks. Third, defects in proteins such as MYO9B that regulate neuronal migration and axon guidance may interfere with the precise positioning of neurons and the accurate extension of axons, thereby contributing to macroscopic brain abnormalities. Fourth, dysfunction of ASPM is predicted to alter the division and differentiation of neural stem cells and to disrupt the proper formation of cortical lamination. Fifth, impaired synaptic vesicle transport due to abnormalities in KIF1A and KIF5C may reduce the efficiency of neurotransmission and compromise information-processing capacity. The convergence of genetic, molecular, and cellular findings suggests that cytoskeletal and molecular motor abnormalities may represent a mechanistic bridge between diverse ASD risk genes and the synaptic and connectivity phenotypes frequently observed in this disorder.

These findings together suggest that ASD is not caused by a single molecular defect but rather arises from a complex interplay among multiple abnormalities in cytoskeletal and molecular motor-related proteins, which collectively perturb neurodevelopment, synaptic function, and network-level information processing.

## 4. Conclusions

Accumulating evidence indicates that abnormalities in cytoskeletal organization and molecular motor function—particularly within the kinesin (KIF) and myosin families—are deeply involved in the pathophysiology of psychiatric disorders such as schizophrenia and ASD. These abnormalities affect multiple aspects of brain function, including neurodevelopment, synapse formation, neurotransmission, and neuronal plasticity, and are thought to contribute to the characteristic symptoms and cognitive impairments observed in these conditions.

Recent findings on the localization and function of Myosin Id in dendritic spines provide important clues for understanding the disease mechanisms of ASD. In our laboratory, we plan to further dissect the roles of Myosin Id and to clarify in greater detail how its dysfunction contributes to ASD onset and progression. In parallel, elucidating the mechanisms by which KIFs transport neurotransmitter receptors and synaptic vesicles —including NMDA receptor subunits whose dysfunction underlies glutamatergic pathophysiology in schizophrenia [72]—has provided new perspectives on the pathogenesis of psychiatric disorders [37]. A more precise understanding of the functions of individual KIFs and their links to specific psychiatric phenotypes is expected to facilitate the development of novel, mechanism-based therapeutic strategies.

Recent large-scale human genetics studies published in the 2020s further reinforce the relevance of synaptic and neurodevelopmental pathways that intersect with cytoskeletal regulation and intracellular transport. For schizophrenia, a large two-stage GWAS reported associations at 287 genomic loci and highlighted convergence on synaptic organization and neuronal differentiation [34]. For ASD, large-scale exome sequencing has identified more than 100 risk genes and emphasized both developmental and functional mechanisms in excitatory and inhibitory neuronal populations [35]. In parallel, recent work has expanded the microglia-focused literature in psychiatric and neurodevelopmental disorders, including evidence linking microglial activation to interneuron metabolic disruptions relevant to schizophrenia [73], reviews integrating microglia–neuron interactions in schizophrenia [74], and functional genomics studies in human iPSC-derived microglia implicating ASD risk genes in endocytosis and synaptic pruning [75].

Growing evidence indicates that microglial cytoskeletal dysfunction is directly implicated in the pathophysiology of both schizophrenia and ASD, rather than representing a generic neuroinflammatory response. In schizophrenia, activated microglia have been shown to cause persistent metabolic disruptions in developing cortical interneurons, including cytoskeletal dysregulation that may underlie the GABAergic circuit abnormalities characteristic of the disorder [73]. A comprehensive review of microglia–neuron interactions in schizophrenia further highlights synaptic pruning dysregulation and altered cytoskeletal dynamics as key contributors to aberrant connectivity [74]. In ASD, a CRISPRi-based screen of ASD risk genes in microglia revealed that ADNP—a microtubule end-binding protein—regulates microglial endocytosis and synaptic pruning, establishing a direct mechanistic link between microglial cytoskeletal function and ASD-associated synaptic pathology [75]. Importantly, the morphological changes accompanying microglial activation involve a shift from acentrosomal to centrosomal microtubule arrays [76] and polarized microtubule remodeling that drives cytokine release [77], providing a mechanistic basis for how cytoskeletal dysfunction in microglia could amplify neuroinflammatory responses relevant to both schizophrenia and ASD. Dysregulated synaptic pruning and altered neuroimmune signaling driven by abnormal microglial cytoskeletal remodeling have been proposed as convergent contributors to aberrant circuit development in both disorders [7,38,64]. Furthermore, upregulation of psychiatric disorder-associated kinesin family members (including KIF3A, KIF17, and KIF13A) in alternatively activated primary cultured microglia [38] suggests that dysregulation of microtubule-based motor transport is not confined to neurons but extends to glial cells, providing a novel mechanistic dimension to the cytoskeletal dysfunction framework in psychiatric disease. Together, these findings highlight the characterization of microglial cytoskeletal and motor protein abnormalities in schizophrenia and ASD as an important priority for future investigation, with potential implications for biomarker development and therapeutic targeting.

Key future directions include:

- Clarifying the relationships between glia-specific cytoskeletal and molecular motor gene variants and psychiatric disorders;
- Developing novel therapeutic strategies that directly target glial cytoskeletal and molecular motor pathways;
- Elucidating the roles of cytoskeletal and motor systems in neuron–glia interactions;
- Defining how morphological changes in glial cells relate to functional alterations in the context of psychiatric illnesses.

Through these lines of investigation, we anticipate further advances in our understanding of the pathophysiological mechanisms underlying psychiatric disorders, which in turn should contribute to the development of new therapeutic and preventive strategies. Approaches that specifically target microglial cytoskeletal and molecular motor dynamics may offer therapeutic benefit for patients with treatment-resistant conditions. Research on cytoskeletal and molecular motor systems lies at the interface of neuroscience and psychiatry and is expected to become increasingly important in the coming years. Ultimately, progress in this field may help improve the quality of life of individuals suffering from psychiatric disorders.

## Figures and Tables

**Table 1:** Cytoskeletal and molecular motor alterations implicated in schizophrenia and ASD, including the type of evidence, reported alterations, molecular functions, and proposed cellular consequences.

Category	Gene/Protein	Type of Evidence	Molecular Function	Reported Alteration	Cellular Consequence	Relevance	Key Refs.
Microtubule	MAP2	Postmortem brain studies	Stabilizes dendritic microtubules	Reduced expression	Dendritic arbor simplification and reduced synaptic integration	Schizophrenia	[39,40]
Microtubule-associated	DISC1	Genetic/functional studies	Regulates microtubule stability and intracellular transport	SNPs: rs821616 (p.Ser704Cys) disrupts NDEL1/LIS1 binding; rs1000731 associated with hippocampal deficits	Neurodevelopmental and synaptic plasticity defects	Schizophrenia	[41,42]
Actin regulation	CYFIP1 (WAVE complex)	CNV/expression studies	Controls actin polymerization in dendritic spines	15q11.2 microdeletion (OR ≈ 2.8 for SCZ); CYFIP1 haploinsufficiency (~50% protein reduction)	Spine dysgenesis and excitatory synapse dysfunction	SCZ/ASD	[43,44,45,46]
Actin regulation	NCKAP1/WASF1	Postmortem expression	Components of WAVE actin regulatory complex	Expression changes	Defective dendritic spine formation	Schizophrenia	[43,44]
Actin-binding	Calponin-3	Proteomics/neuronal assays	Stabilizes actin filaments	Increased expression	Abnormal spine morphology and plasticity	Schizophrenia	[47,48]
Intermediate filament	GFAP	Postmortem brain analysis	Astrocytic cytoskeletal protein	Increased expression	Astrocyte activation and neuroinflammation	Schizophrenia	[49,50]
Kinesin motor	KIF17	Genetics/protein studies	Transports NMDA receptor subunit NR2B	rs2722519 (missense SNP); reduced KIF17 protein expression impairs NR2B-NMDAR trafficking	Impaired NMDA receptor trafficking and synaptic plasticity	Schizophrenia	[51,52]
Kinesin motor	KIF1A	Genetic/rare variants	Anterograde transport of synaptic vesicle precursors	Motor/stalk domain SNPs (SCZ); c.773C > T p.Pro258Leu (ASD, de novo); impaired ATPase/processivity	Reduced neurotransmitter supply to synapses	SCZ/ASD	[53,54,55,56]
Kinesin motor	KIF3B	Mouse model studies	Regulates NMDA receptor trafficking	Kif3b loss-of-function (mouse model); reduces NR2A trafficking, causes SCZ-like behavior	Disrupted NMDA signaling	Schizophrenia	[57]
Dynein motor	DYNC1H1	Rare variant reports	Retrograde axonal transport	De novo missense variants; predicted motor domain destabilization impairing retrograde transport	Impaired trophic signaling and cargo recycling	Schizophrenia	[58,59]
Myosin motor	MYO16	Rare variant studies	Regulates neuronal migration and dendrite formation	Rare missense and CNVs (Han Chinese GWAS); impaired neuronal migration and dendrite formation	Circuit formation abnormalities	SCZ/ASD	[60,61]
Myosin motor	MYO1D	Genetic association	Spine-localized actin motor	Common risk variant (GWAS); TH1-domain–dependent spine localization; excitatory synapse defect	Excitatory synaptic dysfunction	ASD	[17,36]
Myosin metabolism	MYO1D–ASPA	Protein interaction studies	Links cytoskeleton and neuronal metabolism	Common risk variant (GWAS); TH1-domain–dependent spine localization; excitatory synapse defect	Metabolic–synaptic coupling abnormalities	ASD	[62,63]
Myosin motor	MYO9B	Functional neuronal studies	Regulates RhoA signaling and dendritic morphology	Rare missense in RhoGAP domain; impaired RhoA inactivation; dendritic arborization defects	Altered dendritic structure	ASD	[5,64]
PSD scaffold	SHANK3	Human genetics	Organizes actin cytoskeleton in postsynaptic density	22q13.3 deletion (Phelan-McDermid); protein-truncating variants (e.g., p.Arg1117); PSD scaffold loss	Severe dendritic spine defects	ASD	[65,66]
Microtubule nucleation	TUBG1	Rare variant reports	Microtubule nucleation	p.Tyr92Cys (de novo missense); disrupts γ-TuRC; impairs MT nucleation and cortical neuron migration	Neuronal polarity and migration defects	ASD	[67,68]
Neurogenesis regulator	ASPM	Genetic studies	Controls neural progenitor division	Biallelic LoF variants in IQ-domain repeats; symmetric progenitor division defect; ASD cohort CNVs	Cortical development abnormalities	ASD	[69,70]
Kinesin motor	KIF5C	Genetic studies	Axonal transport and neuronal polarity	p.Glu237Lys (de novo, motor domain); ATP hydrolysis impaired; axon elongation and cortical wiring defects	Abnormal axon elongation and circuit wiring	ASD	[71]
Kinesin (microglia)	KIF family (Kifs)	Primary cultured microglia	Intracellular transport in microglia	Upregulation in alternatively activated microglia	Altered microglial transport and immune function	SCZ/ASD	[38]

## Data Availability

No new data were created or analyzed in this study.
